# Influence of Vitamin D Metabolites on Plasma Cytokine Concentrations in Endurance Sport Athletes and on Multiantigen Stimulated Cytokine Production by Whole Blood and Peripheral Blood Mononuclear Cell Cultures

**DOI:** 10.1155/2014/820524

**Published:** 2014-01-01

**Authors:** Cheng-Shiun He, William D. Fraser, Michael Gleeson

**Affiliations:** ^1^School of Sport, Exercise and Health Sciences, Loughborough University, Leicestershire, Loughborough LE11 3TU, UK; ^2^Norwich Medical School, University of East Anglia, Norwich NR4 7TJ, UK

## Abstract

*Aim*. Our aims were to determine the influence of plasma total 25-hydroxy vitamin D (25(OH)D) status on the plasma cytokine concentrations in athletes and the *in vitro* effects of different doses of 1, 25 dihydroxyvitamin D_3_ (1, 25(OH)_2_D_3_) on multiantigen stimulated cytokine production by whole blood and peripheral blood mononuclear cell (PBMC) cultures. *Methods.* Plasma samples from 43 athletes with high and low levels of 25(OH)D were assayed for the concentrations of cytokines. The whole blood samples and PBMCs from healthy subjects were incubated *in vitro* with a multi-antigen vaccine and different doses of added 1, 25(OH)_2_D_3_. The circulating cytokines and stimulated whole blood and PBMC culture production of cytokines were determined using a biochip assay. *Results*. The circulating interleukin-(IL-)10 and interferon-(IFN-) *γ* concentrations were significantly higher in the vitamin D sufficient athletes. Furthermore, the production of tumour necrosis factor-(TNF-) *α*, IL-6, IFN-*γ*, IL-2, and IL-10 by whole blood culture was significantly inhibited by 1, 25(OH)_2_D_3_ concentrations of 1000 pmol/L or 10000 pmol/L. *Conclusions*. We found that the influence of vitamin D on circulating cytokines might be different in athletes compared with nonathletes and cytokines production by whole blood culture was not influenced by 1, 25(OH)_2_D_3_ in concentrations within the normal healthy range.

## 1. Introduction

Vitamin D can be obtained either from dietary sources or the epidermal layer of the skin via exposure to sunlight. The endogenously synthesised vitamin D_3_ and diet-derived D_2_ and D_3_ are hydroxylated in the liver to form 25(OH)D. 25(OH)D is the main storage form, which can be stored in muscles and adipose tissue, and is the major circulating metabolite of vitamin D, with a plasma half-life of 2-3 weeks. Therefore, the plasma concentration of 25(OH)D is considered to be the primary indicator of vitamin D status [1]. Plasma 25(OH)D values commonly accepted as the reference range [2] are as follows. In healthy humans, 25(OH)D > 100 nmol/L are defined as optimal vitamin D status and levels from 50 to 100 nmol/L are defined as adequate. Plasma levels of 25(OH)D < 50 nmol/L are proposed to define inadequate vitamin D status and values < 30 nmol/L represent vitamin D deficiency. Subsequently, 25(OH)D can be converted in the kidney to the biologically active form, 1, 25(OH)_2_D, by 1-*α*-hydroxylase, an enzyme which is stimulated by parathyroid hormone when serum calcium and phosphate concentrations fall below the physiological range. Normal concentrations of circulating 1, 25(OH)_2_D are approximately 50–250 pmol/L, about 1000 times lower than its precursor, 25(OH)D; the plasma half-life of 1, 25(OH)_2_D is 4–6 hours [3].

It has only recently been recognised that vitamin D plays an important role in upregulating immunity [4]. Several recent studies have found a negative association between vitamin D status and respiratory illness incidence in young and elderly adults and it has been indicated that individuals with low vitamin D status have a higher risk of respiratory illness incidence and suffer more severe symptoms when a respiratory illness is present [5–7]. Moreover, the presence of the vitamin D receptor in almost all immune cells, including T lymphocytes, B lymphocytes, neutrophils, and antigen presenting cells, such as macrophages and dendritic cells has been demonstrated and these immune cells also express the vitamin D-activating enzyme, 1-*α*-hydroxylase, and thus possess the ability to convert 25(OH)D to 1, 25(OH)_2_D. This process is regulated by circulating levels of 25(OH)D or induced by activation of specific toll-like receptors which act as detectors of pathogens [1, 8]. Therefore, vitamin D could play vital roles in both innate and adaptive immune responses.

Furthermore, vitamin D may also influence circulating cytokine levels and cytokine production during periods of infection. Within the general population it has been reported that circulating proinflammatory cytokine concentrations, such as TNF-*α*, IFN-*γ*, IL-1*β*, and IL-2, were significantly higher in vitamin D insufficient adults compared with those who were vitamin D sufficient [9]. However, to our knowledge, there has been no study to compare the difference in circulating cytokine levels between vitamin D insufficient and sufficient athletes. Moreover, the anti-inflammatory effects of exercise training [10] might modify the influence of plasma 25(OH)D on circulating cytokine levels in athletes. Thus, it is of interest to determine the influence of plasma 25(OH)D status on circulating cytokine levels in athletes. In addition, several recent studies indicate that 1, 25(OH)_2_D_3_ limits the *in vitro* production of TNF-*α*, IFN-*γ*, and IL-6 by isolated human PBMC stimulated with lipopolysaccharide (LPS) and *Candida albicans *and seasonal variations in vitamin D levels correlate with alterations in cytokine production by *ex vivo* LPS stimulated PBMC, with higher TNF-*α*, IFN-*γ*, and IL-6 production in winter [11, 12]. Nevertheless, in our previous vitamin D study [7], we found that vitamin D deficiency was associated with substantially lower production of proinflammatory cytokines, such as TNF-*α*, IFN-*γ*, IL-1*β*, and IL-6, in the whole blood culture by both monocytes and lymphocytes in response to a multiantigen challenge containing antigens from a virus and both gram-positive and gram-negative bacteria. The reason for this discrepancy is unclear and further research is still needed to understand the influence of vitamin D on multi-antigen stimulated cytokine production.

In the present study, our aims were to determine the influence of plasma 25(OH)D status on the plasma cytokine concentrations of endurance sport athletes and the effects of different doses of 1, 25(OH)_2_D_3_ on the *in vitro* multi-antigen stimulated cytokine production by whole blood and PBMC cultures.

## 2. Methods

### 2.1. Participants

A total of 225 healthy individuals engaged in regular sports training (predominantly endurance-based activities such as running, cycling, swimming, triathlon, team games, and racquet sports) were recruited as subjects from Loughborough University, UK (latitude 53°N), during November 2011, in our previous vitamin D study [7] with the mean age of the study cohort at recruitment being 21 ± 3 years (mean ± SD) and their self-reported training loads averaged 10 h/week. Subjects were required to complete a comprehensive health-screening questionnaire prior to starting the study and had not taken any regular medication or antibiotics in the 3 months prior to the study. All subjects were fully informed about the rationale for the study and of all experimental procedures to be undertaken. Subjects provided written consent to participate in the study, which had earlier received the approval of Loughborough University Ethical Advisory Committee. For the visit to the laboratory, subjects arrived in the morning at 08:30–10:30 following an overnight fast of approximately 12 h and their body mass and height were recorded. Information about the study was given to them and they then signed an informed consent form. Subsequently, a resting venous blood sample (5 mL) was obtained by venepuncture from an antecubital forearm vein into a vacutainer tube (Becton Dickinson, Oxford, UK) containing K_3_EDTA. Haematological analysis was immediately carried out on this sample (including haemoglobin, haematocrit, and total and differential leukocyte counts) using an automated cell-counter (Ac.T 5diff haematology analyser, Beckman Coulter, High Wycombe, UK). Subjects had to have normal haematology to be included in the study. The remaining EDTA blood was centrifuged for 10 min at 1500 g and 4°C and the plasma was stored at −80°C prior to analysis. EDTA plasma samples from 43 athletes with high and low vitamin D status (high level: 25(OH)D > 80 nmol/L, *n* = 18; low level: 25(OH)D < 40 nmol/L, *n* = 25) with sufficient volume were selected for assay of the circulating cytokine concentrations. There was no significant difference in the physical characteristics of participants between high-level and low-level vitamin D status groups (Table 1).

For the study of 1, 25(OH)_2_D_3_ *in vitro* incubation, 6 healthy male individuals (age: 28.8 ± 4.1; height: 176.3 ± 10.0; weight: 83.9 ± 7.8; BMI: 27.1 ± 2.9) who had not taken any regular medication or antibiotics in the 3 months prior to the study were recruited as subjects from Loughborough University. For the visit to the laboratory, subjects arrived in the morning at 08:30–10:30 following an overnight fast of approximately 12 h and subsequently, a resting venous blood sample (20 mL) was obtained by venepuncture from an antecubital forearm vein into vacutainer tubes containing K_3_EDTA and lithium heparin. Haematological analysis was immediately carried out on the EDTA sample using an automated cell counter. Subjects had to have normal haematology to be included in the study. The whole blood samples and the PBMC isolated from the heparin tubes were used immediately for *in vitro* incubation with a multi-antigen vaccine and different doses of 1, 25(OH)_2_D_3_.

PBMC were isolated by density centrifugation on Ficoll-Hypaque, Histopaque 1077 (Sigma Chemicals, Poole, UK). The heparinized whole blood was diluted 1  :  1 with room temperature 10 mM phosphate buffered saline (PBS, Sigma Chemicals, Poole, UK) and 20 mL of diluted blood was layered onto 10 mL of room temperature Ficoll-Hypaque. Samples were then centrifuged at 800 g for 30 minutes at 20°C. Cells were collected from interphase and washed twice with RPMI 1640 medium (Sigma Chemicals, Poole, UK) supplemented with 10% Fetal Bovine Serum (Sigma Chemicals, Poole, UK) and then spun at 250 g for 10 minutes at 20°C. Subsequently, cells were resuspended in RPMI 1640 medium supplemented with 10% Fetal Bovine Serum. Cells were counted in an automated cell counter and the number of cells was adjusted to 2 × 10^6^ cells/mL in RPMI 1640 medium supplemented with 10% Fetal Bovine Serum.

### 2.2. Plasma 25(OH)D Measurements

In our previous vitamin D study [7], 225 EDTA plasma samples were analysed for 25(OH)D_3_ and 25(OH)D_2_ with a high pressure liquid chromatography tandem mass spectrometer (Waters Acuity, Manchester, UK) after a maximum of 10 months in storage with no previous freeze-thaw cycles as described previously [13]. Briefly, 25(OH)D_2_, 25(OH)D_3_, and deuterated internal standard were extracted from plasma samples, following protein precipitation, using isolute C18 solid phase extraction cartridges. Potential interfering compounds were removed by initial elution with 50% methanol followed by elution of the vitamins using 10% tetrahydrofuran in acetonitrile. Dried extracts were reconstituted prior to injection into a high performance liquid chromatography tandem mass spectrometer in the multiple reaction mode (MRM). The MRM transitions (*m*/*z*) used were 413.2 > 395.3, 401.1 > 383.3, and 407.5 > 107.2 for 25(OH)D_2_, 25(OH)D_3_, and hexa-deuterated(OH)D_3_ (internal standard), respectively. Intra-assay CVs were <10% across a working range of 2.5–624 nmol/L for both 25(OH)D_3_ and 25(OH)D_2_. Measurements were performed in a laboratory (Norwich University Hospital, Norwich, UK) meeting the performance target set by the Vitamin D External Quality Assessment Scheme (DEQAS) Advisory Panel for 25(OH)D assays.

### 2.3. *In Vitro* Incubation with 1, 25(OH)_2_D_3_ and Multiantigen Vaccine

The whole blood samples and PBMC were incubated *in vitro* with a multi-antigen vaccine and different doses of 1, 25(OH)_2_D_3_. The stimulant was a commercially available multi-antigen vaccine (Pediacel Vaccine, Sanofi Pasteur, UK) containing diphtheria, tetanus, acellular pertussis, poliomyelitis, and haemophilus influenzae type b antigens. 1, 25(OH)_2_D_3_ was purchased from Sigma-Aldrich (Missouri, USA) and dissolved in absolute ethanol to give a stock solution of 10 *μ*M. Aliquots were subsequently diluted with RPMI 1640 medium for use in the* in vitro* incubations.

Stimulated whole blood and PBMC culture production of cytokines (IFN-*γ*, TNF-*α*, IL-1*β*, IL-2, IL-4, IL-6, and IL-10) were determined as described previously [14]. Briefly, for the determination of baseline unstimulated cytokine production, 0.25 mL of heparinized whole blood or PBMC were added to 0.75 mL of RPMI 1640 medium and incubated at 37°C and 5% CO_2_ for 24 h. Additionally, 0.25 mL of heparinized whole blood or PBMC were added to 0.70 mL of RPMI 1640 medium containing different doses of 1, 25(OH)_2_D_3_ (0, 100, 200, 1000, and 10000 pmol/L) with an added 50 *μ*L of Pediacel vaccine cocktail (Sanofi Pasteur msd Limited, Maidenhead, UK) at a dilution of 1 : 100, before being incubated at 37°C and 5% CO_2_ for 24 h. The stimulant dilution of 1 : 100 used in this study was based on a separate experiment (unpublished data), which established the dose-response curve for the measured cytokines over the dilution range of 1 : 100–1 : 20 000. Samples were then centrifuged at 13000 g for 4 min at 4°C, following which the supernatant fluid was harvested and stored at −80°C prior to analysis of cytokine concentrations.

### 2.4. Cytokine Measurements

Cytokine concentrations were determined using an Evidence Investigator System and the high sensitivity cytokine biochip array EV3513 (Randox, County Antrim, UK). The intra-assay CV for all measured cytokines was less than 5.0%. The measured cytokine concentrations for the monocyte-derived cytokines (TNF-*α*, IL-1*β*, and IL-6) and lymphocyte-derived cytokines (IL-2, IL-4, and IFN-*γ*) were divided by the monocyte and lymphocyte counts, respectively, to give cytokine production per 10^6^ cells.

### 2.5. Statistical Analysis

The independent *t*-test was used to test for the differences in the physical characteristics of participants between high-level and low-level vitamin D groups. Mann-Whitney *U*-test was used to test for the differences in the circulating cytokine concentrations between high-level and low-level vitamin D groups. The correlation between circulating cytokine and plasma 25(OH)D concentrations was determined using Spearman's rank correlation. Wilcoxon signed rank test performed in the previous studies by Khoo and colleagues [11, 12] was also used in the present study to examine for the differences in the stimulated whole blood and PBMC culture production of cytokines between different doses of 1, 25(OH)_2_D_3_ *in vitro* incubation. Differences in the multi-antigen stimulated cytokine production without the addition of 1, 25(OH)_2_D_3_ by whole blood and PBMC cultures were compared with the Mann-Whitney *U* test. Data are presented as mean (±SD) or median and interquartile range (IQR) and the accepted level of significance was *P* < 0.05.

## 3. Results

### 3.1. Circulating Cytokine Concentrations and Vitamin D Status

The plasma 25(OH)D concentrations were 101.3 ± 13.9 and 31.8 ± 3.5 nmol/L in the high-level and low-level vitamin D status groups, respectively. The anti-inflammatory cytokine IL-10 concentration in the high-level vitamin D status group was marginally but significantly higher than in the low-level group (*P* = 0.020), and the proinflammatory cytokine IFN-*γ* concentration in the high-level vitamin D status group was substantially and significantly higher than in the low-level group (*P* = 0.004) (Table 2). However, there was no significant difference in the circulating TNF-*α*, IL-1*β*, IL-2, IL-4, and IL-6 concentrations between high-level and low-level vitamin D status groups (Table 2). In addition, there was a positive correlation between the plasma 25(OH)D and IFN-*γ* concentrations (r = 0.374, P = 0.014), but plasma 25(OH)D concentrations did not correlate with TNF-*α*, IL-1*β*, IL-2, IL-4, IL-6, or IL-10 levels (Table 3).

### 3.2. Antigen-Stimulated Cytokine Production by Whole Blood Culture

**Figure 1:**
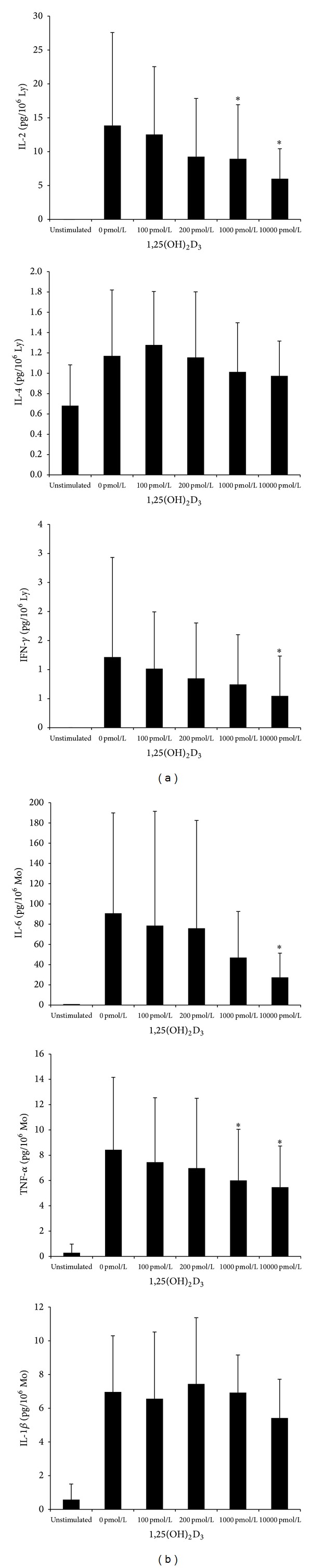
The antigen-stimulated production of the lymphocyte-derived cytokines (IL-2, IL-4, and IFN-*γ* (a)) and the monocyte-derived cytokines (IL-6, TNF-*α*, and IL-1*β* (b)) by whole blood culture. *P < 0.05 versus 0 pmol/L. Ly: lymphocytes; Mo: monocytes.

The antigen-stimulated production of the monocyte-derived cytokines, TNF-*α* and IL-6, was both attenuated by 1, 25(OH)_2_D_3_, but production of IL-1*β* was not significantly influenced by 1, 25(OH)_2_D_3_. The production of TNF-*α* was significantly lower in the presence of 1000 pmol/L and 10000 pmol/L of 1, 25(OH)_2_D_3_ compared with its production without the addition of 1, 25(OH)_2_D_3_ and the production of IL-6 was significantly lower in the presence of 10000 pmol/L of 1, 25(OH)_2_D_3_ compared with its production without the addition of 1, 25(OH)_2_D_3_ (Figure 1). In addition, the antigen-stimulated production of the lymphocyte-derived cytokines, IFN-*γ* and IL-2, was both attenuated by 1, 25(OH)_2_D_3_, but production of IL-4 was not significantly influenced by 1, 25(OH)_2_D_3_. The production of IL-2 was significantly lower in the presence of 1000 pmol/L and 10000 pmol/L of 1, 25(OH)_2_D_3_ compared with its production without the addition of 1, 25(OH)_2_D_3_ and the production of IFN-*γ* was significantly lower in the presence of 10000 pmol/L of 1, 25(OH)_2_D_3_ compared with its production without the addition of 1, 25(OH)_2_D_3_ (Figure 1). The anti-inflammatory cytokine IL-10 is produced by both monocytes and lymphocytes, so it is not appropriate to normalise its production by cell counts. The production of IL-10 was significantly higher in the presence of 10000 pmol/L of 1, 25(OH)_2_D_3_ compared with its production without the addition of 1, 25(OH)_2_D_3_ (0 pmol/L: 1.5 ± 0.8 pg/mL; 10000 pmol/L: 4.5 ± 2.1 pg/mL; *P* = 0.028), but no differences were observed at the other concentration of added 1, 25(OH)_2_D_3_.

### 3.3. Antigen-Stimulated Cytokine Production by PBMC Culture

**Figure 2:**
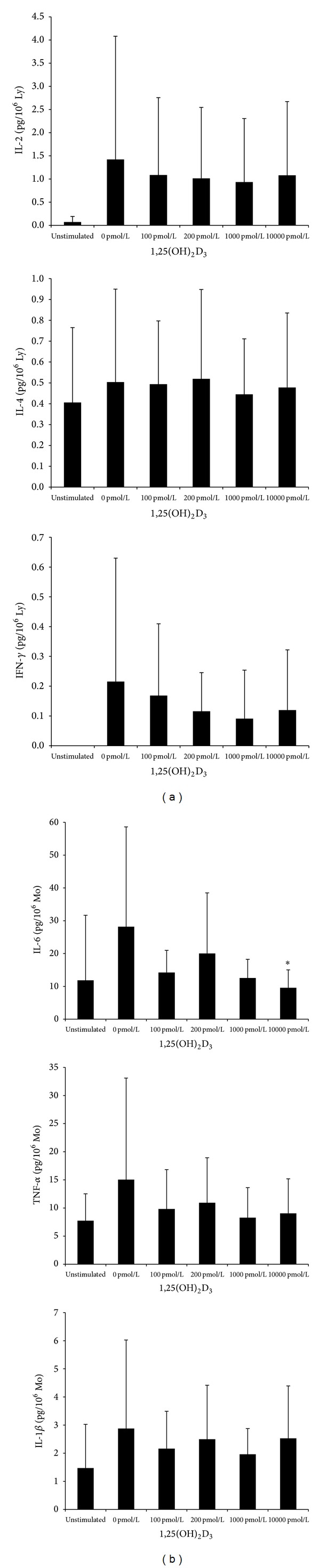
The antigen-stimulated production of the lymphocyte-derived cytokines (IL-2, IL-4, and IFN-*γ* (a)) and the monocyte-derived cytokines (IL-6, TNF-*α*, and IL-1*β* (b)) by PBMC culture. *P < 0.05 versus 0 pmol/L. Ly: lymphocytes; Mo: monocytes.

The antigen-stimulated production of the monocyte-derived cytokine, IL-6, was attenuated by 1, 25(OH)_2_D_3_, but production of TNF-*α* and IL-1*β* was not significantly influenced by 1, 25(OH)_2_D_3_. The production of IL-6 was significantly lower in the presence of 10000 pmol/L of 1, 25(OH)_2_D_3_ compared with its production without the addition of 1, 25(OH)_2_D_3_ (Figure 2). However, the antigen-stimulated production of the lymphocyte-derived cytokines, IFN-*γ*, IL-2, and IL-4, was not significantly influenced by the incubation of different doses of 1, 25(OH)_2_D_3_ (Figure 2). In addition, the production of IL-10 was also not significantly influenced by 1, 25(OH)_2_D_3_.

### 3.4. Comparison of the Whole Blood and PBMC Culture

The production of the lymphocyte-derived cytokines, IFN-*γ*, IL-2, and IL-4, and the monocyte-derived cytokine, IL-1*β*, by multi-antigen stimulated whole blood culture without the addition of 1, 25(OH)_2_D_3_ was significantly higher than their respective production by the PBMC culture (Table 4). However, there was no significant difference in the antigen-stimulated production of TNF-*α*, IL-6, and IL-10 between the whole blood and PBMC cultures (Table 4).

## 4. Discussion

The aims of this research were to determine the influence of plasma 25(OH)D status on the circulating cytokine levels in endurance sport athletes and the effects of different doses of 1, 25(OH)_2_D_3_ on the multi-antigen stimulated cytokine production by whole blood and PBMC cultures. The main findings of the present study were that the circulating IL-10 and IFN-*γ* concentrations were significantly higher in the vitamin D sufficient athletes compared with the vitamin D insufficient athletes and there was a positive correlation between the plasma 25(OH)D and IFN-*γ* concentrations in endurance athletes. Furthermore, the production of TNF-*α*, IL-6, IFN-*γ*, IL-2, and IL-10 by multi-antigen stimulated whole blood culture was significantly influenced by 1, 25(OH)_2_D_3_ of concentrations of 1000 pmol/L and/or 10000 pmol/L. Nevertheless, a similar effect was only observed for the production of IL-6 by multi-antigen stimulated PBMC culture. In addition, the production of lymphocyte-derived and monocyte-derived cytokines, such as IFN-*γ*, IL-2, IL-4, and IL-1*β*, was significantly different in the multi-antigen stimulated whole blood culture without the addition of 1, 25(OH)_2_D_3_ compared with its production in the PBMC culture.

On the basis of the present data, we found that the circulating IL-10 and IFN-*γ* concentrations in the vitamin D sufficient athletes were significantly higher than in the vitamin D insufficient athletes and there was a positive correlation between the plasma 25(OH)D and IFN-*γ* concentrations in endurance athletes. In contrast, a previous study has reported an elevation of circulating proinflammatory cytokines in vitamin D insufficient adults [9]. Barker et al. [9] indicated that circulating proinflammatory cytokine concentrations, such as TNF-*α*, IFN-*γ*, IL-1*β*, and IL-2, were significantly higher in vitamin D insufficient compared to vitamin D sufficient adults but the anti-inflammatory cytokine, IL-10, was not significantly different between vitamin D insufficient and sufficient adults. Moreover, Barker and colleagues [9] found an inverse correlation between the plasma 25(OH)D and IFN-*γ* concentrations. The reason for this discrepancy is still unclear but may be due to the difference of the cut-off point used for vitamin D insufficiency in the two studies. In healthy humans, plasma 25(OH)D serum levels > 100 nmol/L are defined as optimal vitamin D status and levels from 50 to 100 nmol/L are defined as adequate. Serum levels of 25(OH)D < 50 nmol/L are proposed to define inadequate vitamin D status and values < 30 nmol/L represent vitamin D deficiency [2]. Therefore, the commonly used cut-off point for vitamin D insufficiency in clinical practice and research reports is the threshold concentration of 25(OH)D of < 50 nmol/L [15]. However, the cut-off point for vitamin D insufficiency used in the Barker et al. [9] study was < 80 nmol/L and the average serum 25(OH)D concentration of vitamin D of insufficient adults was around 60 nmol/L. Thus, the vitamin D insufficient adults in their study were not even close to being vitamin D deficient. In addition, another confounding factor may be due to the anti-inflammatory effects of exercise training. The participants recruited in our previous vitamin D study [7] were the endurance sport athletes engaged in regular sports training (predominantly endurance-based activities such as running, cycling, swimming, triathlon, team games, and racquet sports) and their self-reported training loads averaged 10 h/week. The anti-inflammatory effects of exercise training on cytokine responses, such as the release of IL-6 from contracting muscle and the increased levels of circulating IL-10, cortisol, and adrenaline, are discussed in the review article by Gleeson et al. [10]. It has been demonstrated that the active skeletal muscle significantly increases both cellular and circulating levels of IL-6 during and following exercise of sufficient load and the transient rise in circulating IL-6 during exercise appears to be responsible for a subsequent rise in circulating levels of anti-inflammatory cytokines, including IL-10, which is a potent promoter of an anti-inflammatory state [10]. Increases in circulating cortisol and adrenaline during exercise are due to activation of the hypothalamic-pituitary-adrenal axis and the sympathetic nervous system, respectively. Cortisol is known to have potent anti-inflammatory effects and also augmented by the rise in circulating IL-6 from contracting skeletal muscle [10]. Therefore, the influence of vitamin D on circulating cytokines might be different between athletes and nonathletes. Further research is still needed to understand the influence of vitamin D on circulating cytokines between athletes and nonathletes.

The finding of higher circulating IFN-*γ* concentrations in the vitamin D sufficient athletes and a positive correlation between the plasma 25(OH)D and IFN-*γ* concentrations in the present study might be helpful to support the arguments that vitamin D could play a role in reducing both the severity and duration of upper respiratory tract illness symptoms in our previous vitamin D study [7]. It has been reported that increasing IFN-*γ*, in the circulation contributes to the conversion of 25(OH)D to its active hormonal form, 1, 25(OH)_2_D, in the circulation during inflammatory stress in humans [16]. 1, 25(OH)_2_D has a vital role in upregulating the production of antimicrobial proteins and peptides, such as cathelicidin and *β*-defensin, which are produced by both epithelial cells and macrophages and have a broad range of activities against microorganisms including the direct inactivation of viruses [17] and the hormone also acts to maintain a balance between inflammatory Th1/Th17 cells and immunosuppressive Th2/regulatory T cells to temper inflammation and tissue damage [18]. Therefore, the vitamin D sufficient athletes with higher circulating IFN-*γ* concentrations may have less severe symptoms and shorter duration when the upper respiratory tract illness is present.

According to the findings from the present study, it seems likely that the multi-antigen stimulated cytokine production by whole blood culture was influenced by 1, 25(OH)_2_D_3_ only in concentrations that were 1000 pmol/L or more. Although the production of proinflammatory cytokines, TNF-*α*, IL-6, IFN-*γ* and IL-2 by multi-antigen stimulated whole blood culture was significantly lower in the presence of 1000 pmol/L or 10000 pmol/L of 1, 25(OH)_2_D_3_ and the production of anti-inflammatory cytokine, IL-10, was significantly higher in the presence of 10000 pmol/L of 1, 25(OH)_2_D_3_, we found that both proinflammatory and anti-inflammatory cytokine production by multi-antigen stimulated whole blood culture were not influenced by 1, 25(OH)_2_D_3_ in added concentrations of 100 pmol/L and 200 pmol/L. In contrast, the results from our previous study [7] showed that vitamin D status (determined by plasma levels of 25(OH)D) did significantly influence lymphocyte and monocyte proinflammatory cytokine production by antigen-stimulated whole blood culture. Individuals with high levels of plasma 25(OH)D would be expected to have higher levels of 1, 25(OH)_2_D, so it is perhaps surprising that in the *in vitro* situation variations in 1, 25(OH)_2_D within the normal physiological range appear to have no significant effect on leukocyte cytokine production. In healthy humans, normal concentrations of circulating 1, 25(OH)_2_D are approximately 50–250 pmol/L, about 1000 times lower than its precursor, 25(OH)D [3]. Inhibition of proinflammatory cytokine production and elevation of anti-inflammatory cytokine production in the present study were only observed when the whole blood was incubated with 1, 25(OH)_2_D in concentrations that were 10–100-fold above the normal healthy range for plasma 1, 25(OH)_2_D. Therefore, the physiological relevance is unclear. A limitation of the present study is that the participants' plasma concentration of 1, 25(OH)_2_D was not determined. Further research is still needed to understand the influence of the normal healthy range of plasma 1, 25(OH)_2_D on the multi-antigen stimulated cytokine production by whole blood culture.

The data from our study show that there was a significant difference in the production of multi-antigen stimulated lymphocyte-derived and monocyte-derived cytokines, such as IFN-*γ*, IL-2, IL-4, and IL-1*β*, between the whole blood and PBMC cultures and it seems likely that there was a better dose-dependent response in the cytokine production of multi-antigen stimulated whole blood culture with different doses of 1, 25(OH)_2_D_3_ than in the PBMC culture. The whole blood culture retains the normal cellular, hormonal, and cytokine milieu that the leukocytes are normally exposed to in the circulation. This model probably comes closest to the natural environment avoiding artefacts from cell isolation and preparation and allowing natural interactions between immune components and antigens within the normal hormonal milieu. Essentially it is an *in vitro* method of simulating responses to an infection. Moreover, the stimulant we used was a vaccine containing antigens from a virus and both gram-positive and gram-negative bacteria and the multiple antigen challenge used in the present study provides valuable information on cytokine production since not all cytokines respond to the same antigen. The capacity of leukocytes to produce cytokines upon adequate challenge (e.g., with mitogen, antigen, endotoxin, or pathogen exposure) has potentially far reaching consequences for the entire functional capacity of the immune system. Therefore, the whole blood culture stimulated with multi-antigen is highly likely to reflect the capacity of an individual to defend itself against intruding microorganisms.

In conclusion, the present study demonstrated that the influence of vitamin D on circulating cytokines might be different in athletes compared with previous studies on nonathletes and the higher circulating IFN-*γ* concentrations in vitamin D sufficient athletes might be helpful to support the arguments that vitamin D could play a role in reducing both the severity and duration of upper respiratory tract illness symptoms. In addition, we found that both proinflammatory and anti-inflammatory cytokines by multi-antigen stimulated whole blood culture were not influenced by 1, 25(OH)_2_D_3_ in concentrations within the normal healthy range. Furthermore, the whole blood culture stimulated with multi-antigen might be a better *in vitro* method of simulating responses to an infection than cultures using PBMCs.

## Figures and Tables

**Table 1:** Physical characteristics of 43 athletes with high and low vitamin D status.

	High-level vitamin D	Low-level vitamin D	*P*
Number (M : F)	18 (8 : 10)	25 (19 : 6)	
Age (years)	20.9 ± 4.1	19.9 ± 2.0	0.294
Height (cm)	174.0 ± 10.1	177.0 ± 8.5	0.309
Weight (kg)	71.0 ± 13.5	73.3 ± 9.4	0.516
BMI (kg/m^2^)	23.3 ± 2.8	23.3 ± 1.8	0.960
Mean training loads (Mean MET-h/wk)	84.9 ± 46.2	64.0 ± 28.2	0.100

Data are mean ± SD.

M: male; F: female.

*P* value from independent *t*-test is shown in right hand column.

**Table 2:** Circulating cytokine concentrations between high-level and low-level vitamin D status groups.

	High-level vitamin D	Low-level vitamin D	*P*
IL-2 (pg/mL)	3.8 (2.6–4.5)	2.5 (0.0–5.4)	0.186
IL-4 (pg/mL)	1.9 (1.8–2.3)	1.8 (1.5–2.0)	0.127
IL-6 (pg/mL)	1.3 (1.0–1.9)	0.9 (0.7–1.5)	0.218
IL-10 (pg/mL)	0.7 (0.6–1.6)	0.6 (0.5–0.8)	0.020
IFN-*γ* (pg/mL)	0.9 (0.6–1.4)	0.5 (0.0–0.7)	0.004
TNF-*α* (pg/mL)	2.6 (1.8–3.2)	2.4 (2.0–3.3)	0.825
IL-1*β* (pg/mL)	4.5 (3.3–5.7)	3.9 (2.8–7.2)	0.912

Data are median and IQR.

*P* value from Mann-Whitney *U*-test is shown in right hand column.

**Table 3:** The correlation between circulating cytokine and plasma 25(OH)D concentrations.

	IL-2	IL-4	IL-6	IL-10	IFN-*γ*	TNF-*α*	IL-1*β*
Spearman's rank correlation	0.179	0.197	0.126	0.257	0.374	−0.157	0.006
*P* value	0.250	0.206	0.421	0.097	0.014	0.314	0.970

**Table 4:** Comparison of the whole blood and PBMC culture in antigen-stimulated cytokine production.

	Whole blood	PBMC	*P*
IL-2 (pg/10^6^ Ly)	9.5 (4.5–16.6)	0.3 (0.2–0.7)	0.016
IL-4 (pg/10^6^ Ly)	1.0 (0.7–1.6)	0.4 (0.3–0.4)	0.025
IL-6 (pg/10^6^ Mo)	48.9 (16.5–145.3)	15.4 (12.1–23.5)	0.262
IL-10 (pg/mL)	1.7 (1.5–1.8)	2.0 (1.6–2.3)	0.297
IFN-*γ* (pg/10^6^ Ly)	0.6 (0.3–0.9)	0.1 (0.0–0.1)	0.037
TNF-*α* (pg/10^6^ Mo)	8.4 (3.6–11.9)	7.7 (6.4–11.7)	0.631
IL-1*β* (pg/10^6^ Mo)	6.4 (4.3–8.8)	1.6 (1.3–2.6)	0.037

Data are median and IQR.

Ly: lymphocytes; Mo: monocytes.

*P* value from Mann-Whitney *U*-test is shown in right hand column.
